# Higher intraocular pressure is associated with slower axial growth in children with non-pathological high myopia

**DOI:** 10.1038/s41433-023-02872-7

**Published:** 2023-12-11

**Authors:** Fabian SL Yii, Mingguang He, Francesca Chappell, Miguel O. Bernabeu, Tom MacGillivray, Baljean Dhillon, Andrew Tatham, Niall Strang

**Affiliations:** 1 Centre for Clinical Brain Sciences, The University of Edinburgh, Edinburgh, UK; 2 Curle Ophthalmology Laboratory, Institute for Regeneration and Repair, The University of Edinburgh, Edinburgh, UK; 3 School of Optometry, The Hong Kong Polytechnic University, Hong Kong, China; 4 State Key Laboratory of Ophthalmology, Zhongshan Ophthalmic Center, Sun Yat-sen University, Guangzhou, China; 5 Centre for Eye Research Australia, The University of Melbourne, Melbourne, Australia; 6 Centre for Medical Informatics, Usher Institute, The University of Edinburgh, Edinburgh, UK; 7 The Bayes Centre, The University of Edinburgh, Edinburgh, UK; 8 Princess Alexandra Eye Pavilion, NHS Lothian, Edinburgh, UK; 9 Department of Vision Sciences, Glasgow Caledonian University, Glasgow, UK

**Keywords:** Risk factors, Prognostic markers

## Abstract

**Objectives:**

To investigate the association between intraocular pressure (IOP) and axial elongation rate in highly myopic children from the ZOC-BHVI High Myopia Cohort Study.

**Methods:**

162 eyes of 81 healthy children (baseline spherical equivalent: −6.25 D to −15.50 D) aged 7–12 years with non-pathological high myopia were studied over five biennial visits. The mean (SD) follow-up duration was 5.2 (3.3) years. A linear mixed-effects model (LMM) was used to assess the association between IOP (at time point *t−1*) and axial elongation rate (annual rate of change in AL from *t−1* to *t*), controlling for a pre-defined set of covariates including sex, age, central corneal thickness, anterior chamber depth and lens thickness (at *t−1*). LMM was also used to assess the contemporaneous association between IOP and axial length (AL) at *t*, controlling for the same set of covariates (at *t*) as before.

**Results:**

Higher IOP was associated with slower axial growth (*β* = −0.01, 95% CI −0.02 to −0.005, *p* = 0.001). There was a positive contemporaneous association between IOP and AL (*β* = 0.03, 95% CI 0.01–0.05, *p* = 0.004), but this association became progressively less positive with increasing age, as indicated by a negative interaction effect between IOP and age on AL (*β* = −0.01, 95% CI −0.01 to −0.003, *p* = 0.001).

**Conclusions:**

Higher IOP is associated with slower rather than faster axial growth in children with non-pathological high myopia, an association plausibly confounded by the increased influence of ocular compliance on IOP.

## Introduction

Building on decades of work using animal models of eye growth, it is now established that axial myopia arises from altered scleral biomechanics [1]. This is manifested anatomically by increased scleral creep rate (i.e. increased tissue extensibility under a constant load) and axial elongation in response to the distending force of intraocular pressure (IOP) [1]. A logical extension of this schema of myopia pathophysiology, which has long garnered attention, is whether raised IOP per se could promote myopia progression [2]. This prospect is not only implicated by substantive evidence from cross-sectional studies that reported higher IOP in myopic eyes [3–7], but also strengthened by recent genetic evidence that pointed towards a causal association between IOP and myopia [8].

Longitudinal studies investigating the association between IOP and myopia progression, however, are scarce. Controversy remains as to whether higher IOP is associated with faster myopia progression: two studies [9, 10] reported an association, but with conflicting directions of correlation, whereas one [11] reported no such association. While differences in study population (e.g. derived from clinical trial versus school-based screening programme) and study design may partly account for these discrepancies, it has been suggested that such association may be more pronounced among high myopes on account of their more compliant sclera [12]. To that end, we investigated if higher IOP was associated with faster axial growth in children with non-pathological high myopia.

## Methods

### Study participants

Longitudinal data from participants enroled in the ZOC-BHVI High Myopia Cohort Study in China (commenced in 2011) were used in the present study. The protocol (including power calculation) has been described in detail elsewhere [13]. In brief, participants with bilateral high myopia (at least −6.00 D spherical power in both eyes) were recruited from the community-based optometry clinic of Zhongshan Ophthalmic Center, which saw close to 300,000 patients each year. Exclusion criteria included secondary (syndromic) myopia, history of previous refractive surgery, autoimmune diseases (e.g. systemic lupus erythematosus) and severe systemic health conditions (e.g. kidney failure). The study adhered to the principles of the Declaration of Helsinki and ethics approval (2012KYNL002) was obtained from the Ethics Committee of Zhongshan Ophthalmic Center. Written informed consent was obtained and signed by each participant.

Of the 222 eyes of 111 children aged 7–12 years at baseline, both eyes of 29 children (58 eyes) were excluded from the present study due to the presence of pathologic myopia in at least one eye (27 right eyes and 24 left eyes had diffuse chorioretinal atrophy). Pathologic myopia was defined based on the Meta Analysis of Pathologic Myopia framework, i.e. myopic maculopathy equal to or more severe than diffuse chorioretinal atrophy and/or the presence of any of the three ‘plus’ signs including lacquer cracks, Fuchs spot and myopic choroidal neovascularization [14]. Two trained ophthalmologists who were masked to the severity of myopia graded each fundus photograph independently. A senior ophthalmologist (MH) acted as an adjudicator in the event of disagreement. One participant with bilateral retinitis pigmentosa was further excluded, leaving 162 eyes of 81 children with non-pathological high myopia to be analysed. These children were followed up at approximately 2-yearly intervals. Data from 60, 50, 43 and 31 children were available at the first (around 2 years after baseline visit), second, third and fourth follow-up, respectively.

### Ocular measurements

Axial length (AL), central corneal thickness (CCT), anterior chamber depth (ACD) and lens thickness (LT) were measured with an optical low-coherence reflectometer (Lenstar LS900, Haag-Streit AG, Koeniz, Switzerland). Three IOP readings were taken during clinic opening hours under topical anaesthesia with a Goldmann applanation tonometer by a trained nurse, and their median was used for subsequent analyses. Objective refraction was measured under cycloplegia (two drops of 0.5% tropicamide administered 5 min apart) with a KR8800 autorefractor (Topcon Corporation, Tokyo, Japan), followed by subjective refraction by an optometrist.

### Statistical analysis

A linear mixed-effects model (with random intercepts) estimated using restricted maximum likelihood was first used to assess the contemporaneous association between IOP at a given time point (*t*) and AL (dependent variable) at the same time point, controlling for a pre-defined set of covariates including age, sex, CCT, ACD and LT. The model can be expressed as follows:

1 $${{AL}}_{t}={{IOP}}_{t}+{{age}}_{t}+{{IOP}}_{t}\times {{age}}_{t}+{sex}+{{CCT}}_{t}+{{ACD}}_{t}+{{LT}}_{t}+{Intercept}$$

The subscript *t*, which is appended to time-varying variables, denotes a specific time point (i.e. one of the five visits), while the interaction term ${{IOP}}_{t}\times {{age}}_{t}$ examines if the relationship between ${{AL}}_{t}$ and ${{IOP}}_{t}$ changes according to ${{age}}_{t}$. Another LMM was used to investigate the association between IOP at a preceding time point (*t−1*) and the amount of axial growth from *t−1* to *t* divided by the difference in age between these time points (annual axial elongation rate at *t*, ${{Rate}}_{t}$). The model adjusted for the same set of covariates as before at *t−1*. It can be expressed as follows:

2 $${{Rate}}_{t}	= {{IOP}}_{t-1}+{{age}}_{t-1}+{{IOP}}_{t-1}\times {{age}}_{t-1}+{sex}\\ 	 +{{CCT}}_{t-1}+{{ACD}}_{t-1}+{{LT}}_{t-1}+{Intercept}$$

The interaction term ${{IOP}}_{t-1}\times {{age}}_{t-1}$ examines if the effect of IOP at *t−1* on the annual axial elongation rate at *t* changes according to the age at *t−1*.

Both regression models used the eye as the unit of analysis to increase statistical power [15]. The inter-eye correlation and repeated measures correlation were accounted for by treating eyes as random effects nested within individuals [15, 16]. IOP and all continuous covariates were mean-centred in both models so that the intercept corresponded to the AL or annual axial elongation rate of a ‘typical’ individual, i.e. when IOP and other continuous covariates were equal to their respective mean values. Multicollinearity was checked with the variance inflation factor, treating ten as the cut-off value [17]. Error normality and homogeneity of variance were checked by examining the normal probability and Residuals versus Fits plots, which indicated that both assumptions were not violated. All analyses were performed using R Statistical Software (v4.2.2; R Core Team 2022).

## Results

### Baseline characteristics

Table 1 summarises the baseline characteristics of the 81 included children, consisting of 38 males and 43 females with a mean (SD) age of 10.1 (1.5) years. The mean (SD) follow-up duration was 5.2 (3.3) years. Paired *t* tests showed no statistical differences between eyes except for LT, although the difference was practically irrelevant (0.007 mm before rounding). Eight children had missing baseline IOP in both eyes. Compared to those with IOP data, these children were younger (8.4 vs 10.3 years old, unpaired t-test: *p* = 0.003), but they did not differ in terms of sex distribution (chi-square test: *p* = 0.71) and other baseline characteristics (*p* > 0.05 in all unpaired *t* tests).

**Table 1:** Baseline characteristics of the included eyes.

	Right eye				Left eye				*P*
	*N*_eyes_	Mean	SD	Range	*N*_eyes_	Mean	SD	Range	
SER (D)	81	−8.64	1.88	−6.25 to −15.25	81	−8.89	2.06	−6.25 to −15.50	0.08
AL (mm)	80	26.69	0.88	24.78 to 29.14	80	26.74	0.81	25.13 to 29.19	0.33
IOP (mmHg)	73	15.71	2.52	11 to 23	73	15.63	2.30	11 to 22	0.47
CCT (μm)	80	545.1	30.0	476 to 610	80	546.1	30.4	476 to 611	0.12
ACD (mm)	80	3.23	0.23	2.51 to 3.71	80	3.22	0.22	2.54 to 3.69	0.38
LT (mm)	80	3.38	0.15	3.00 to 3.76	80	3.38	0.16	3.01 to 3.79	0.03

Mean differences between right and left eyes were tested with paired t-tests.

*N*_eyes_: number of eyes with available data.

*SER* spherical equivalent refraction, *AL* axial length, *IOP* intraocular pressure, *CCT* central corneal thickness, *ACD* anterior chamber depth, *LT* lens thickness

### Contemporaneous association between IOP and AL

**Fig. 1:**
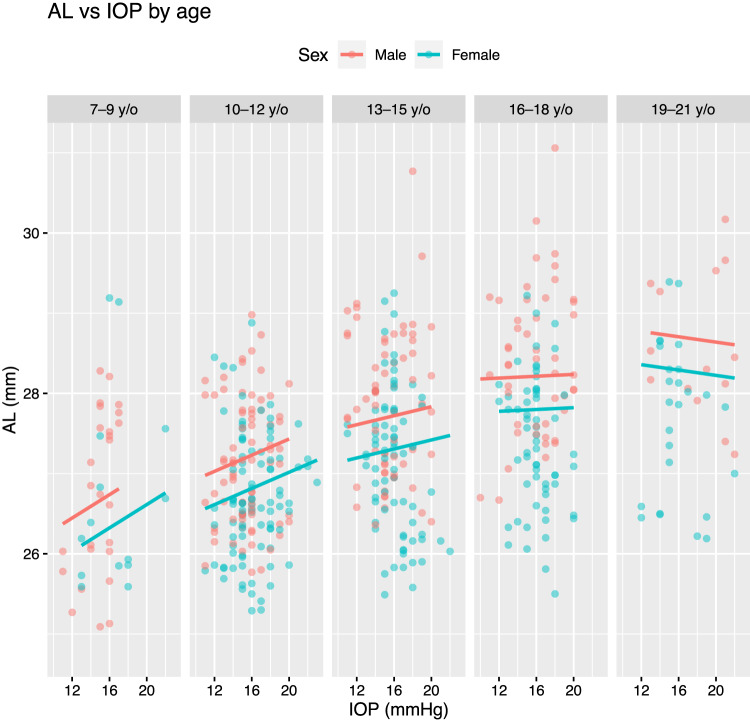
Contemporaneous association between intraocular pressure, IOP (at time point *t*) and axial length, AL (similarly at *t*) by age group per the fitted multivariable model presented in Table 2. Regression lines are plotted using 8 years old (y/o), 11 y/o, 14 y/o, 17 y/o and 20 y/o to represent the 7–9 y/o, 10–12 y/o, 13–15 y/o, 16–18 y/o and 19–21 y/o age groups, respectively.

From Table 2, it can be seen that every 1 mmHg increase in IOP was associated with 0.03 mm greater AL (95% CI: 0.01 mm to 0.05 mm) while holding covariates including age, ACD and sex constant, i.e. setting continuous covariates to their respective mean values and sex to male. AL also increased with age, with a 0.16 mm (95% CI: 0.15 mm to 0.18 mm) increase in AL observed for each year of increasing age. There was a negative interaction effect between IOP and age on AL, such that the positive association between IOP and AL became progressively less positive as age increased (Fig. 1). This interaction effect (while holding other covariates constant) was given by:

$${AL}=0.03\times \left({IOP}-{{Mean}}_{{IOP}}\right)-0.01 \times ({IOP}-{{Mean}}_{{IOP}}) \times ({Age}-{{Mean}}_{{age}})$$

Where ${{Mean}}_{{IOP}}$ and ${{Mean}}_{{age}}$ correspond to 16 mmHg and 13 years, respectively. To illustrate, at age 7, every 1 mmHg increase in IOP was associated with 0.09 mm increase in AL; conversely, the same increase in IOP was associated with 0.02 mm decrease in AL at age 18. Females were found to have 0.41 mm smaller AL on average, although there was considerable uncertainty around this estimate (95% CI: 0.80 mm smaller AL to 0.03 mm greater AL).

**Table 2:** Contemporaneous association between IOP and AL (dependent variable) per linear-mixed effects model (1), controlling for age, sex, CCT, ACD and LT.

Dependent variable: AL at time point *t* (mm)						
IV & covariates at t	Mean	*N*	VIF	Estimate	95% CI	*P*
Intercept	NA	NA	NA	27.62	27.35 to 27.90	<0.001
IOP (per mmHg)	16	503	1.1	0.03	0.01 to 0.05	0.004
Age (per year)	13	522	2.6	0.16	0.15 to 0.18	<0.001
IOP × Age	NA	501	1.1	−0.01	−0.01 to −0.003	0.001
CCT (per μm)	549	522	1.1	0.00	−0.003 to 0.01	0.48
ACD (per mm)	3.2	522	2.1	0.81	0.10 to 1.51	0.03
LT (per mm)	3.5	522	4.1	0.68	−0.17 to 1.53	0.12
Sex: Female	NA	288	1.1	−0.41	−0.80 to 0.03	0.04
Sex: Male	NA	236	Reference			

IV: Independent variable (IOP at time point *t*).

N: Number of observations where IV or covariate and AL are available.

VIF: Variance inflation factor.

*AL* axial length, *IOP* intraocular pressure, *CCT* central corneal thickness, *ACD* anterior chamber depth, *LT* lens thickness.

### Influence of IOP on axial elongation rate

**Fig. 2:**
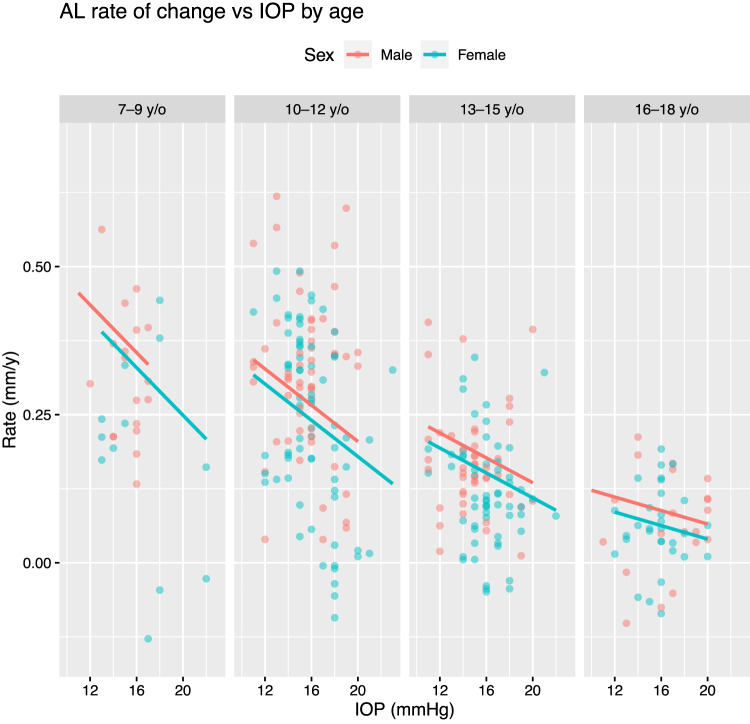
Association between intraocular pressure, IOP (at time point *t* − 1) and annual change in axial length, AL from *t* − 1 to t (separated by 2 years on average) by age group per the fitted multivariable model presented in Table 3. Regression lines are plotted using 8 years old (y/o), 11 y/o, 14 y/o and 17 y/o to represent the 7–9 y/o, 10–12 y/o, 13–15 y/o and 16–18 y/o age groups, respectively.

A negative independent association between IOP and annual axial elongation rate over subsequent longitudinal follow-up was observed. Every 1 mmHg increase in IOP at *t-1* was predicted to slow axial growth by 0.01 mm/y (95% CI: −0.02 mm/y to −0.005 mm/y) from *t* *−* *1* to *t* (Table 3 and Fig. 2). Axial growth was also predicted to slow by 0.03 mm/y (95% CI: 0.03 mm/y to 0.02 mm/y slower), on average, with every year increase in age. There was insufficient evidence of an interaction effect between IOP and age on axial elongation rate. However, the regression coefficient of the interaction term (equal to 0.002 before rounding) suggests that the negative association between IOP and axial elongation rate gradually became more positive (slope tended towards zero) as age increased, which could plausibly be attributed to an attenuation of axial growth with older age (Fig. 2). None of the other covariates were found to influence axial elongation rate, except CCT, although only a weak positive association was noted, i.e. every 1 μm increase in CCT was expected to increase AL growth rate by just 0.001 mm/y (95% CI: 4 × 10^−5 ^mm/y to 0.002 mm/y).

**Table 3:** Association between IOP and annual axial elongation rate over subsequent follow-up (dependent variable) per linear mixed-effects model (2), controlling for age, sex, CCT, ACD and LT.

Dependent variable: annual axial growth from time point *t−1* to *t* (mm/y)						
IV and covariates at *t* − 1	Mean	*N*	VIF	Estimate	95% CI	*P*
Intercept	NA	NA	NA	0.20	0.16 to 0.23	<0.001
IOP (per mmHg)	16	313	1.1	−0.01	−0.02 to −0.005	0.001
Age (per year)	13	326	1.4	−0.03	−0.03 to −0.02	<0.001
IOP × Age	NA	313	1.1	0.00	0.00 to 0.00	0.08
CCT (per μm)	549	326	1.2	0.00	0.00 to 0.00	0.05
ACD (per mm)	3.2	326	1.4	0.08	−0.03 to 0.19	0.18
LT (per mm)	3.5	326	1.6	−0.10	−0.25 to 0.04	0.17
Sex: Female	NA	184	1.1	−0.03	−0.08 to 0.03	0.34
Sex: Male	NA	142	Reference			

IV: independent variable (IOP at time point *t*−1).

*N*: number of observations where IV or covariate and axial elongation rate are available.

VIF: variance inflation factor.

*IOP* intraocular pressure, *CCT* central corneal thickness, *ACD* anterior chamber depth, *LT* lens thickness.

## Discussion

In our cohort of children with non-pathological high myopia (mean baseline SER around −8.75 D), higher IOP was associated with slower axial growth over subsequent follow-up. Consistent with the results from previous cross-sectional studies [3–7], we also found a positive contemporaneous association between IOP and AL, but this association became progressively less positive with increasing age.

Recent evidence from Mendelian randomisation (MR), which provides strong genetic causal inference on account of its robustness to confounding, suggests a positive bidirectional causal association between IOP and myopia [8]. There is also strong evidence of a shared genetic basis for myopia and primary open-angle glaucoma [18]. One may, therefore, infer that raised IOP fundamentally promotes faster axial elongation—and that a greater amount of axial elongation also leads to a larger increase in IOP, perhaps due to myopia-related structural alterations that influence the uveoscleral outflow pathway (e.g. thickened ciliary body) [19, 20]. In light of this, how can one reconcile our finding of a negative association between IOP and axial growth rate with the seemingly contradictory inference drawn from MR?

The prevailing theory of myopic eye growth implicates a biochemical signalling cascade that ultimately increases scleral compliance [1]. Anatomically, the cornea and sclera are closely linked and undergo similar stromal changes in response to myopia [21]. This, along with indirect evidence from previous studies, has led to the notion that corneal biomechanics may reflect whole-eye biomechanics. For instance, Taroni et al. [22] found that human eyes with stiffer sclera induced by scleral buckling had lower in vivo corneal hysteresis (CH) than the non-operated fellow eyes. Ex vivo studies also demonstrated that stiffened sclera from human donor eyes exhibited increased resistance to corneal deformation (stiffer cornea) [23–25]. Importantly, recent evidence suggests that the cornea also becomes more compliant or less stiff in eyes with high myopia. For example, in vivo human studies employing Corvis ST (OCULUS, Wetzlar, Germany)—which is a dynamic tonometer that allows the reaction of the cornea to a defined air pulse to be recorded using a high-speed Scheimflug camera—consistently found evidence of increased corneal deformation (e.g. higher maximal deformation amplitude, larger peak distance, increased concavity of the deformation) [26–28] upon applanation in highly myopic eyes. Moreover, using a specially designed corneal indentation device, Hon et al. [29] observed a reduction in corneal resistance to indentation (lower elastic modulus) in human eyes with high axial myopia (AL > 26 mm). A similar observation was reported by an in vitro study looking at chick eyes with experimentally induced high myopia [30].

In addition to its association with myopia severity, corneal compliance/stiffness has also been shown to influence Goldmann IOP measurement. Liu et al. [31] demonstrated that the normal variation in corneal elastic modulus alone could give rise to as much as 17.26 mmHg difference in Goldmann IOP, where an increase in corneal compliance (other things being equal) would cause IOP to be underestimated. In line with this, an increase in corneal compliance following cataract surgery has been found to be accompanied by a reduction in Goldmann IOP, even after adjustment for changes in CCT [32]. Liu et al. [33] also reported an increase in IOP following in vitro stiffening of porcine cornea using glutaraldehyde solution while maintaining other geometrical and material properties of the eye. This is further illustrated by our observation that the initial positive (contemporaneous) association between IOP and AL—measured at a time when the children in our study had lower myopia with less compliant eyes—became increasingly negative when they grew older, as the corresponding increase in myopia and ocular compliance would be expected to cause IOP to be increasingly underestimated.

Taken together, our intriguing finding that higher Goldmann IOP was associated with slower (rather than faster) axial growth could be attributed to the confounding effect of corneal compliance on IOP measurement in high myopes. We suggest that above a certain AL threshold characterised by pronounced ocular compliance, the fundamental (i.e. causal, as inferred from MR) positive association between IOP and axial elongation rate becomes increasingly masked by this confounding effect, such that Goldmann IOP becomes increasingly indicative of ocular compliance and less reflective of the true IOP. A high IOP reading obtained from an eye with high axial myopia, in short, may be indicative of stiffer sclera, which in turn means that the eye concerned is less susceptible to axial elongation. This hypothesis may also explain why, among young children with low to moderate myopia (e.g. baseline SER not more myopic than −4.50D or −5.75D)—a level at which ocular compliance is not likely to be a significant confounder—a positive or no significant association between baseline IOP and myopia progression rate was generally found by previous longitudinal studies [9, 11].

Our hypothesis that the confounding effect of ocular compliance/stiffness is implicated in the observed association between higher IOP and slower axial growth, however, is weakened somewhat by Wan et al. [34]. The authors of that study found that lower CH was associated with faster axial growth in children aged 6–10 years. Although CH should not be directly equated with stiffness or compliance, as it is a composite measure of both viscosity and elasticity [35], a lower CH accompanied by higher Goldmann IOP is known to have some association with increased corneal stiffness [36]. If this is so, one should expect lower CH to be associated with slower rather than faster axial growth based on our hypothesis. Having said that, this contradiction might arise from the use of children with much lower baseline myopia (none had an SER < −4.50 D) in Wan et al. [34], which, understandably, might have a different ocular biomechanical profile from our highly myopic children. Instead of viewing CH as a surrogate for scleral stiffness, Wan et al. [34] postulated that the posterior tissue of eyes with lower CH had reduced ability to dissipate the distending force of IOP, rendering them more susceptible to axial elongation. Ultimately, the question of how ocular biomechanics might be involved in the association between IOP and axial growth in eyes with different levels of myopia remains an open question until direct investigation of the interaction between IOP and in vivo measurement of scleral biomechanics becomes possible. Until the nature of this interaction is understood, perhaps using a more sophisticated statistical technique such as structural equation modelling [37], any hypothesis remains a matter of conjecture.

The main strengths of the present study are its longitudinal design and the use of a generally healthy cohort of children with non-pathological high myopia, a group known to have significantly altered scleral biomechanics [38]. This enabled us to specifically assess if axial elongation in eyes with high myopia might be affected differently (and in what respect) by IOP, an investigation hitherto not attempted by any other study. The present study also employed Goldmann applanation tonometry, which is the gold standard for IOP measurement. However, our study was limited by a lack of standardisation of the time at which IOP was measured. As such, the effect of diurnal variation in IOP could not be accounted for [39]. Moreover, the use of an ethnically homogeneous (Chinese) cohort in the present study may not generalise to other ethnic groups. Our relatively modest sample size may also limit the precision of the estimate of the effect size of IOP, although this was mitigated to some extent by our choice of using the eye (rather than individual) as the unit of analysis. Last but not least, it is important to bear in mind that the present study, despite its longitudinal design, neither provides evidence for nor against causality (and its direction), which is a question that MR is designed to answer.

In conclusion, higher IOP was associated with slower rather than faster axial elongation in eyes of children with non-pathological high myopia. While the clinical science underpinning IOP physiology, ocular biomechanics and eye growth is complex and much remains to be elucidated, it seems plausible that Goldmann IOP is, to some extent, indicative of ocular biomechanics in high axial myopia. If so, their dynamic interaction is likely to be an important determinant of pathology implicating ocular biomechanics, notably pathologic myopia. A better understanding of these dynamics could prove to be useful in better targeting high myopes at risk of such pathology.

## Summary

### What was known before

- Intraocular pressure (IOP) increased with increasing levels of myopia, but longitudinal studies investigating the influence of IOP on myopia progression are scarce.
- High axial myopia is characterised by pronounced scleral compliance which may render the eye more amenable to the distending force of IOP.

### What this study adds

- In children with non-pathological high myopia, higher Goldmann IOP was associated with slower rather than faster axial growth.
- This association could plausibly be explained by the increased influence of ocular compliance on IOP (i.e., IOP measurements are increasingly reflective of ocular biomechanics with increasing myopia).
- Future studies could investigate the independent prognostic value of Goldmann IOP in identifying high axial myopes at risk of pathologic myopia.

## Data Availability

The dataset is available upon reasonable request (contact Prof Mingguang He).
